# Cortical bone loss is an early feature of nonradiographic axial spondyloarthritis

**DOI:** 10.1186/s13075-018-1620-1

**Published:** 2018-08-30

**Authors:** Anna Neumann, Judith Haschka, Arnd Kleyer, Louis Schuster, Matthias Englbrecht, Andreas Berlin, Camille P. Figueiredo, David Simon, Christian Muschitz, Roland Kocijan, Heinrich Resch, Jürgen Rech, Georg Schett

**Affiliations:** 1 Department of Internal Medicine 3, Friedrich Alexander University Erlangen-Nurnberg and Universitätsklinikum Erlangen, Ulmenweg 18, 91054 Erlangen, Germany; 2 St. Vincent Hospital, VINFORCE Study Group, Medical University of Vienna, Vienna, Austria; 3 Division of Rheumatology, Faculdade de Medicina da Universidade de São Paulo, São Paulo, Brazil

**Keywords:** Spondyloarthritis, Bone loss, Computed tomography

## Abstract

**Background:**

In the present study, we investigated bone geometry, microstructure, and volumetric bone mineral density (vBMD) in a cohort of patients with nonradiographic axial spondyloarthritis (nr-axSpA) in order to define the early bone changes occurring in axial spondyloarthritis (axSpA) and to define potential factors for deterioration of bone microstructure.

**Methods:**

Patients with axSpA (*n* = 107) and healthy control subjects (*n* = 50) of similar age and sex were assessed for geometric, volumetric, and microstructural parameters of bone using high-resolution peripheral quantitative computed tomography (HR-pQCT) at the radius. Additionally, demographic and disease-specific characteristics of patients with axSpA were recorded.

**Results:**

Patients with nr-axSpA and control subjects were comparable in age, sex, and body mass index. Geometric and microstructural analysis by HR-pQCT revealed a significantly reduced cortical area (*p* = 0.022) and cortical thickness (*p* = 0.006) in patients with nr-axSpA compared with control subjects. Total and cortical vBMD were significantly reduced in patients with nr-axSpA (*p* = 0.042 and *p* = 0.007, respectively), whereas there was no difference in trabecular vBMD. Patients with a short disease duration (< 2 years; *n* = 46) also showed significant reduction of cortical thickness and cortical area compared with control subjects. Patients with disease duration > 2 years (*n* = 55) additionally developed a decrease of cortical and total vBMD. Multiple regression models identified male sex to be associated with lower cortical vBMD and female sex to be associated with lower trabecular vBMD.

**Conclusions:**

Bone microstructure in patients with nr-axSpA is characterized primarily by deterioration of cortical bone. Cortical bone loss starts early and is evident within the first 2 years of the disease.

**Electronic supplementary material:**

The online version of this article (10.1186/s13075-018-1620-1) contains supplementary material, which is available to authorized users.

## Background

Spondyloarthritis (SpA) comprises a group of diseases with shared genetic and pathophysiologic backgrounds affecting the axial and peripheral skeleton. Axial disease comprises nonradiographic axial SpA (nr-axSpA) and ankylosing spondylitis (AS). Axial inflammation leads to local bone formation with progressive ankylosis of sacroiliac joints as well as syndesmophyte formation. Nonetheless, systemic bone loss and increased vertebral and nonvertebral fracture risk have been described in AS [1].

Authors of a recent review of the literature on bone mass in AS reported a prevalence of osteoporosis varying from 3% to 47% according to different measurement techniques and patient selection criteria, whereas osteopenia has been reported in up to 88% of patients [2]. These variations may be based on the fact that bone analysis in nr-axSpA and AS is challenging, particularly in the axial skeleton, where it is confounded by local new bone formation. Especially in patients with syndesmophytes, dual-energy X-ray absorptiometry (DXA) is unreliable because it sums up new bone formation with bone loss owing to its two-dimensional nature [3, 4]. However, bone loss has also been found at the hip of patients with AS [5–8]. Furthermore, quantitative computed tomography (QCT), which separately measures trabecular and cortical volumetric bone density, has supported the occurrence of bone loss in AS, showing reduced trabecular volumetric bone mineral density (vBMD) in the lumbar spine in severe AS with syndesmophyte formation [9].

Systemic inflammation is at least partly responsible for systemic bone loss in patients owing to proinflammatory cytokines leading to a direct activation of osteoclastogenesis [10]. In prior studies, patients with AS showed significant bone loss as measured by DXA at the lumbar spine compared with patients with mechanical back pain [11]. Further, in the DESIR (DEvenir des Spondyloarthrites Indifférenciées Récentes) cohort, 71.4% of patients with inflammatory back pain fulfilled Assessment of Spondyloarthritis international Society (ASAS) classification criteria for axial spondyloarthritis (axSpA), and in multiple logistic regression analyses, bone marrow edema seen on magnetic resonance imaging scans, markers of inflammation such as C-reactive protein (CRP) and erythrocyte sedimentation rate (ESR), and male sex were associated with lower bone mineral density (BMD) at any site [12]. Inhibition of tumor necrosis factor (TNF)-α has been reported to show a beneficial effect on BMD and bone turnover markers of patients with AS, thereby supporting the role of systemic inflammation on bone metabolism [13–15]. In accordance with this, vertebral fractures in patients with AS are associated with the longer disease duration independent of age [16].

Bone strength depends not only on BMD but also on bone geometry and microarchitectural aspects of cancellous and cortical bone [17, 18]. High-resolution quantitative computed tomography (HR-pQCT) permits a noninvasive, three-dimensional assessment of bone geometry, volumetric BMD, and microarchitecture, which resembles a virtual bone biopsy of peripheral bone [19, 20]. HR-pQCT data have been shown to correlate with BMD results obtained by DXA and with incident fracture risk in the radius, hip, and spine in postmenopausal women [21, 22].

The aim of the present study was to investigate bone microstructure, geometry, and vBMD using HR-pQCT in a large cohort of patients with nr-axSpA in early stages of disease and to search for potential risk factors for deterioration of bone microstructure. In this context, it is important to mention that patients with AS with long-standing disease are characterized by reduction of total and cortical vBMD and cortical thickness, as well as increased cortical porosity [23, 24]. Data on early disease, however, which may reflect the initial changes of bone in nr-axSpA, are missing to date.

## Methods

### Patients and control subjects

A total of 107 Caucasian patients with a diagnosis of nr-axSpA were recruited at the Department of Internal Medicine 3 of the University of Erlangen-Nuremberg, Germany. All patients fulfilled the ASAS classification criteria. Patients with AS were not included in this study because we were specifically seeking to study a population with early bone changes. Recruitment of healthy control subjects has been described elsewhere, and these individuals were matched by age and sex [25]. Briefly, exclusion criteria for being a healthy control subject were (1) presence or history of chronic joint pain/swelling, (2) presence of systemic diseases, (3) documented osteopenia/osteoporosis, (4) present or past use of bisphosphonates or prednisolone, and (5) positivity for anticitrullinated protein antibodies (ACPA) or rheumatoid factor. This study was approved by the local ethics committee and the national radiation safety agency (Bundesamt fur Strahlenschutz). Informed consent was obtained from each patient, and the study was performed in accordance with the Declaration of Helsinki.

### Demographic and disease-specific characteristics

Demographic characteristics, disease duration, features of SpA (psoriasis, inflammatory bowel disease [IBD], uveitis), human leukocyte antigen (HLA)-B27 status, ESR, and serum CRP levels were recorded in all patients. Current disease activity was determined by Ankylosing Spondylitis Disease Activity Score (ASDAS-CRP; inactive disease ASDAS-CRP < 1.3, 1.3–2.0 moderate disease activity, 2.1–3.5 high disease activity, > 3.5 very high disease activity). Spinal mobility was assessed by Bath Ankylosing Spondylitis Metrology Index (BASMI). For the assessment of present enthesitis, the Maastricht Ankylosing Spondylitis Enthesitis Score was used. Peripheral arthritis was assessed by 78 tender and 76 swollen joint counts. The Spondylitis Disease Activity Index (BASDAI) was assessed as a secondary disease activity measure, and the Bath Ankylosing Spondylitis Functional Index (BASFI) was used for patient-reported outcomes.

The use of conventional disease-modifying antirheumatic drugs (methotrexate, sulfasalazine, azathioprine, leflunomide, chloroquine, gold) and biologic agents (TNF inhibitors [TNFi]) was recorded. Further, the current or previous use of systemic glucocorticoid (GC) treatment exceeding continuous treatment with 5 mg of prednisolone-equivalent daily for more than 3 months was assessed. Treatment with nonsteroidal anti-inflammatory drugs was also recorded (on demand, daily). History of nontraumatic fractures, diagnosis of osteoporosis, and previous or ongoing antiresorptive treatment or supplementation of 25(OH)vitamin D_3_ and calcium was taken.

### High-resolution peripheral quantitative computed tomography

HR-pQCT imaging was performed in all patients and 50 healthy age- and sex-matched control subjects using the XtremeCT scanner (SCANCO Medical, Brüttisellen, Switzerland). The scan region was selected according to the manufacturer’s standard in vivo protocol of the ultradistal radius of the dominant hand. For measurement, the patient’s hand was immobilized using a carbon fiber shell to reduce movement. Standardization of measurements was ensured by daily cross-calibrations with a standardized control phantom (QRM, Moehrendorf, Germany). The ROI was determined with an anteroposterior scout view and was fixed 9.5 mm proximal from the reference line. The effective dose for each scan was < 3 μSv. The reference line was set manually. The scan ROI was examined in 110 parallel slices (82-μm voxel size) with a total measurement time of 2.8 minutes. All measurements and evaluations were performed using the manufacturer’s standard software. Motion grading (1–5) of each scan was performed using SCANCO Medical standard operating procedure scale, and scans graded > 3 were excluded from analysis.

vBMD, bone microstructure, and geometry were measured with HR-pQCT. Three-dimensional vBMD of the total radius (total BMD in mg of hydroxyapatite [HA]/cm^3^), the cortical shell (Ct. BMD, mg HA/cm^3^), and the trabecular compartment (Tb.BMD, mg HA/cm^3^) were extracted. Additional, distinctive results of trabecular BMD adjacent to bone cortex (mg HA/cm^3^) and central medullary trabecular BMD (mg HA/cm^3^) were expressed. Results of bone microstructure included bone volume fraction (BV/TV, %), trabecular number (mm^−1^), trabecular thickness (μm), trabecular separation (Tb.Sp, μm), inhomogeneity of the trabecular network (μm), cortical thickness (Ct.Th, μm), and cortical porosity (Ct.Po, %). Furthermore, bone geometry was represented by total, cortical, and trabecular bone area (mm^2^). All these parameters were calculated by using automated software. Reliability of the automated contouring method of the Xtreme CT scanner software has recently been shown [26].

### Statistical analysis

Data were collected, organized, and analyzed using IBM SPSS Statistics software (IBM, Armonk, NY, USA). If not stated otherwise, categorical variables are presented as number and percent, and continuous variables are provided as median (IQR). Inferential comparisons comprised chi-square tests for categorical variables (indicated as number and percent in the tables) to check for observed deviations from expected frequencies, as well as the Kruskal-Wallis and Mann-Whitney *U* tests to compare data derived from interval scales. To investigate potential relationships of total, cortical, and trabecular vBMD with disease-related or demographic parameters, multiple linear regression models were computed with an enter procedure including all predictors at a single step. The first model incorporated sex, age, BMI, and smoking status. The second model included sex, age, BMI, remission status, disease duration, treatment with TNFi, prior GC treatment, HLA-B27 status, and peripheral arthritis. A *p* value less than 0.05 was considered significant.

## Results

### Characteristics of patients with nr-axSpA and healthy control subjects

A total of 107 patients with nr-axSpA and 50 healthy control subjects were recruited for this bone analysis. Six patients with nr-axSpA could not be further analyzed, owing to unacceptable motion artefacts, leaving 101 patients with nr-axSpA to be analyzed. Demographic and disease-specific characteristics are shown in Table 1. Patients with nr-axSpA and control subjects were comparable in age (median [IQR], 45.0 [15.0] vs. 44.76 [26.0] years, *p* = 0.917), sex (females, 41.6% vs. 40%, *p* = 0.852), and BMI (median [IQR], 26.3 [6.5] vs. 23.8 [5.2], *p* = 0.118). Of the patients, 37.6% were former or current smokers, with no significant difference compared with healthy control subjects.

**Table 1:** Demographic and disease-specific characteristics of patients with nonradiographic axial spondyloarthritis and healthy control subjects

	Nr-axSpA (*n* = 101)	Control subjects (*n* = 50)	*p* Value
Demographic characteristics			
Female sex, *n* (%)	42 (41.6)	20 (40)	0.852
Age, yr	45.0 (15.0)	44.76 (26.0)	0.917
Height, m	1.74 (0.1)	1.74 (0.1)	0.997
Weight, kg	81.1 (21.5)	76.0 (18.5)	0.054
Body mass index, kg/m^2^	26.3 (6.5)	23.8 (5.2)	0.118
Current or previous smoking, *n* (%)	38 (37.6)	11 (24.4)	0.119
Disease-specific characteristics			
HLA-B27 positivity, *n* (%)	75 (75.0)	–	–
Duration of disease, yr	6.5 (9.0)	–	–
Disease remission, *n* (%)	15 (14.9)	–	–
ASDAS-CRP, units	2.1 (1.4)	–	–
BASDAI, units	3.5 (3.6)	–	–
C-reactive protein, mg/L	6.2 (4.0)	–	–
ESR, mm	12.7 (10.5)	–	–
BASFI, units	2.9 (3.8)	–	–
BASMI, units	1.1 (2)	–	–
Peripheral arthritis, *n* (%)	35 (34.7)	–	–
Dactylitis, *n* (%)	2 (2.0)	–	–
Enthesitis, *n* (%)	17 (16.8)	–	–
MASES, units	1.1 (1)	–	–
Psoriasis, *n* (%)	13 (12.9)	–	–
Uveitis, *n* (%)	8 (7.9)	–	–
Inflammatory bowel disease, *n* (%)	7 (6.9)	–	–
Low trauma fracture, *n* (%)	7 (6.9)	–	–
25(OH)vitamin D_3_, ng/ml	32.4 (16.3)	–	–
Treatment modalities			
Current biologic therapy^a^, *n* (%)	59 (58.4)	–	–
Duration of biologic therapy, yr	2.0 (4.0)		
Current DMARD therapy^b^, *n* (%)	28 (27.7)	–	–
NSAID, *n* (%)	68 (67.3)	–	–
NSAID daily, *n* (%)	22 (21.8)	–	–
NSAID on demand, *n* (%)	46 (45.5)	–	–
Prednisolone ≥ 5 mg > 3 months^c^, *n* (%)	32 (31.7)	–	–
Calcium substitution, *n* (%)	8 (7.9)	–	–
25(OH)vitamin D_3_ substitution, *n* (%)	26 (25.7)	–	–
Antiresorptive treatment, *n* (%)	5 (5.0)	–	–

*Abbreviations: axSpA* Axial spondyloarthritis, *ESR* Erythrocyte sedimentation rate, *25(OH)vitamin D*_*3*_ 25-Hydroxyvitamin D_3_, *ASDAS-CRP* Ankylosing Spondylitis Disease Activity Score, defined as inactive < 1.3, moderate < 2.1, high < 3.5, very high > 3.5 disease activity, disease remission defined as ASDAS-CRP < 1.3, *BASDAI* Bath Ankylosing Spondylitis Disease Activity Index, *BASFI* Bath Ankylosing Spondylitis Functional Index, *BASMI* Bath Ankylosing Spondylitis Metrology Index, *MASES* Maastrich Ankylosing Spondylitis Enthesitis Score, *DMARD* Disease-modifying antirheumatic drug, *NSAID* Nonsteroidal anti-inflammatory drug

Results are median (IQR) or absolute value and percent

^a^Tumor necrosis factor inhibitors

^b^Methotrexate, sulfasalazine, azathioprine, leflunomide, mesalazine

^c^History of treatment with ≥ 5 mg prednisolone for ≥ 3 months

Of patients with nr-axSpA, 75% showed HLA-B27 positivity, and 12.9% of the patients had psoriasis, 7.9% had anterior uveitis, and 6.9% had IBD in their medical history. Systemic inflammation markers were only minimally elevated, with CRP at 6.2 (4.0) mg/L and ESR at 12.7 (10.5) mm. Median disease duration was 6.5 (9.0) years. Disease activity as assessed by ASDAS-CRP was 2.1 (1.4). Only 14.9% of patients were in ASDAS-CRP remission, despite 58.4% of patients being on TNFi treatment with a median (IQR) duration of 2.0 (4.0) years of treatment. Details on the characteristics of patients with nr-axSpA receiving a TNFi and those without a TNFi are summarized in Additional file 1. Clinical assessment by BASMI showed mild impairment of spinal mobility with a median (IQR) score of 1.1 (2) units, whereas patient-reported outcomes revealed a BASDAI of 3.5 (3.6) units and a BASFI of 2.9 (3.8).

Serum 25(OH)vitamin D_3_ level (median (IQR)) was 32.4 (16.3) ng/ml. Fractures after inadequate trauma were reported in seven patients (6.9%), five patients had prior or current antiresorptive treatment, 7.9% had supplementation with calcium, and 25.7% had supplementation with 25(OH)vitamin D_3_.

### Bone geometry and vBMD in patients with nr-axSpA

Bone geometry showed a significant difference in the cortical area between patients with nr-axSpA and control subjects (*p* = 0.022), whereas there was no difference in the total bone area (*p* = 0.700) or the trabecular area (*p* = 0.374) (Table 2). Cortical vBMD suggested a reduction in patients with axSpA (compared with control subjects; *p* = 0.007), whereas trabecular BMD showed no difference (*p* = 0.376). Also, no difference between nr-axSpA and control subjects was found for the central medullary and peripheral trabecular density adjacent to cortex (*p* = 0.310 and *p* = 0.941, respectively). Overall total vBMD was different in patients with axSpA, showing lower values than in control subjects (*p* = 0.042), which was based on the differences in cortical BMD.

**Table 2:** Bone microstructure in patients with nonradiographic axial spondyloarthritis assessed by high-resolution peripheral quantitative computed tomography

	axSpA (*n* = 101)	Control subjects (*n* = 50)	*p* Value
Bone geometry			
Total bone area, mm^2^	334 (108)	326 (116)	0.700
Ct. area, mm^2^	60 (20)	65 (23)	**0.022**
Tb. area, mm^2^	265 (78)	254 (83)	0.374
Volumetric bone mineral density			
Total BMD, HA/cm^3^	313 (70)	334 (61)	**0.042**
Ct. BMD, HA/cm^3^	823 (66)	846 (78)	**0.007**
Tb. BMD, HA/cm^3^	174 (44)	183 (68)	0.376
Tb. meta BMD, HA/cm^3^	234 (43)	243 (61)	0.310
Tb. inn BMD, HA/cm^3^	133 (47)	141 (72)	0.491
Bone microstructure			
BV/TV, %	14.5 (3.7)	15.2 (5.7)	0.383
Tb. N, mm^−1^	2.08 (0.31)	2.11 (0.42)	0.799
Tb. Th, μm	70 (15)	72 (17)	0.486
Tb. Sp, μm	417 (83)	410 (104)	0.602
Inhomogeneity, μm	172 (44)	169 (47)	0.828
Ct. Th, μm	767 (190)	840 (160)	**0.006**
Ct. Po, %	2.4 (1.66)	2.2 (1.57)	0.685

*Abbreviations: axSpA* Axial spondyloarthritis, *Ct*. Cortical, *Tb*. Trabecular, *Tb. meta BMD* Peripheral trabecular density adjacent to cortex, *Tb. inn BMD* Central medullary trabecular density, *BV/TV* Trabecular bone volume, *Th* Thickness, *Sp* Separation, *Po* Porosity

Bone geometry, microstructure, and volumetric bone mineral density (BMD) determined by high-resolution peripheral quantitative computed tomography at the ultradistal radius. Results are median (interquartile range)

Bold indicates significant differences (*p* < 0.05)

### Bone microstructure in patients with nr-axSpA

**Fig. 1:**
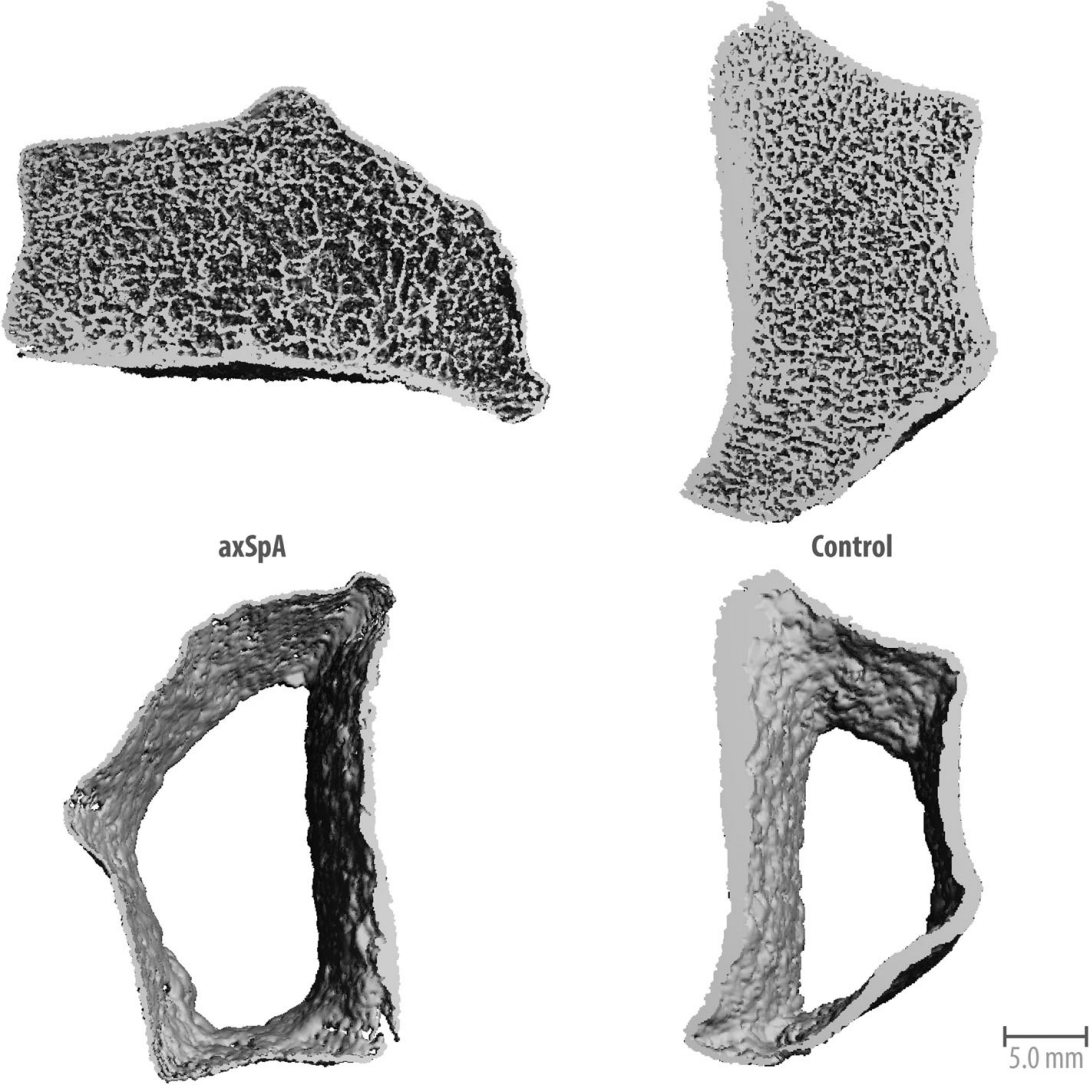
High-resolution peripheral quantitative computed tomographic scans of the ultradistal radius of patients with axial spondyloarthritis (axSpA) and healthy control subjects. Three-dimensional reconstruction of the cortical bone of the total scan region of patients with axSpA and healthy control subjects displays cortical thinning in patients with spondyloarthritis

**Fig. 2:**
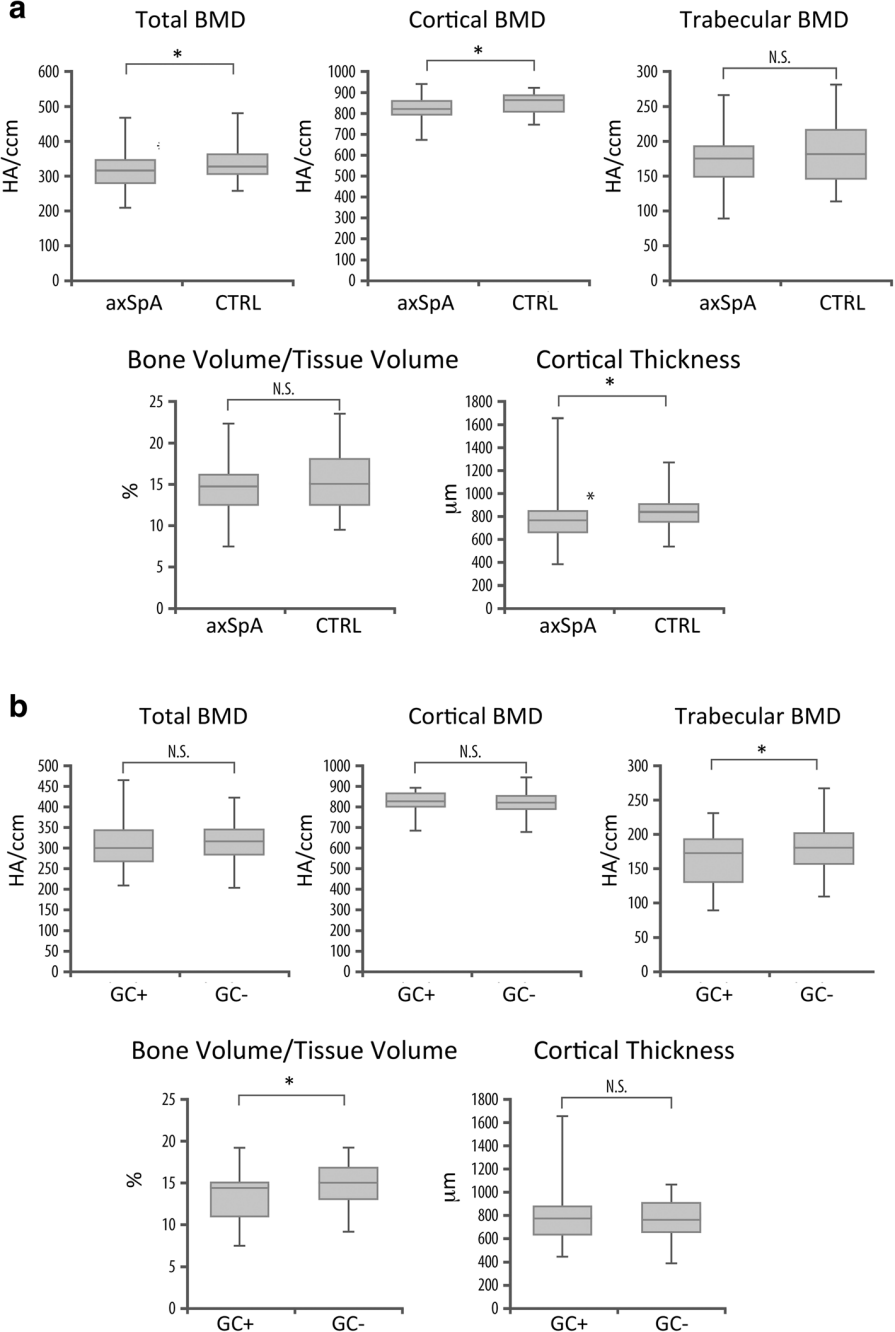
Differences in bone microarchitecture in patients with nonradiographic axial spondyloarthritis (nr-axSpA). **a** Changes of total, cortical, and trabecular bone mineral density (BMD) and bone microarchitecture reflected by total bone volume (BV/TV) and cortical thickness (Ct.Th) between patients with axial spondyloarthritis (SpA) and healthy control subjects. **b** Changes of total, cortical, and trabecular BMD and bone microarchitecture reflected by BV/TV and Ct.Th between patients with nr-axSpA with or without a history of treatment with > 5 mg prednisolone equivalent for more than 3 months. *GC* Glucocorticoids, *N.S.* Not significant, *HA* Hydroxyapatite. *p <  0.05

Assessment of parameters of bone microstructure showed results very consistent with bone geometry and vBMD with reduced cortical thickness (*p* = 0.006). Trabecular bone structure showed no reduction in trabecular bone volume (*p* = 0.383), number (*p* = 0.799), thickness (*p* = 0.486), or increased inhomogeneity (*p* = 0.828) or trabecular separation (*p* = 0.602). Further cortical analysis showed no increases in cortical porosity (*p* = 0.685) or in pore volume and pore diameter (*p* = 0.919 and *p* = 0.827, respectively). (Figs. 1 and 2a).

### Bone microarchitecture and vBMD with respect to disease duration in patients with nr-axSpA

The overall disease duration was 6.5 (9) years, with men having longer disease duration than women (5.0 [10] vs. 2.0 [4], *p* = 0.021). To assess the impact of disease duration on bone microstructure and vBMD, patients were divided into two groups. Forty-six patients had a disease duration of less than 2 years. Comparison between the two groups and healthy control subjects revealed a significant difference in cortical vBMD (*p* = 0.012), cortical thickness (*p* = 0.015), and cortical pore diameter (*p* = 0.007). No difference in trabecular bone density and microstructure was observed (Table 3). Further intergroup comparisons showed that cortical vBMD is decreased in patients with long-standing disease compared with control subjects (*p* = 0.004), whereas there was only a trend in early SpA (*p* = 0.096). Cortical thickness, however, is already decreased in early nr-axSpA (*p* = 0.050), but the decrease is more prominent in patients with long-standing disease than in control subjects (*p* = 0.007).

**Table 3:** Bone microstructure in patients with nonradiographic axial spondyloarthritis with short and longer disease duration and in healthy control subjects

	axSpA < 2 yr (*n* = 46)	axSpA > 2 yr (*n* = 55)	Control subjects (*n* = 50)	axSpA < 2 yr vs. axSpA > 2 years vs. control subjects	axSpA < 2 yr vs. control subjects	axSpA < 2 yr vs. axSpA > 2 yr	axSpA > 2 yr vs. control subjects
Bone geometry							
Total bone area, mm^2^	317 (114)	349 (94)	326 (116)	0.060	0.322	**0.023**	0.136
Ct. area, mm^2^	59 (18)	61 (22)	65 (23)	0.068	**0.032**	0.703	0.066
Tb. area, mm^2^	250 (95)	277 (88)	254 (83)	0.068	0.628	**0.041**	0.062
Volumetric bone mineral density							
Total BMD, HA/cm^3^	317 (49)	309 (75)	334 (61)	0.096	0.170	0.423	**0.036**
Ct. BMD, HA/cm^3^	833 (62)	814 (65)	846 (78)	**0.012**	0.096	0.167	**0.004**
Tb. BMD, HA/cm^3^	173 (44)	175 (47)	183 (68)	0.571	0.289	0.564	0.610
Tb. meta BMD, HA/cm^3^	233 (38)	234 (51)	243 (61)	0.561	0.294	0.728	0.465
Tb. inn BMD, HA/cm^3^	132 (47)	135 (52)	141 (72)	0.618	0.359	0.464	0.753
Bone microstructure							
BV/TV, %	14.4 (3.7)	14.6 (3.9)	15.2 (5.7)	0.575	0.288	0.564	0.628
Tb. N, mm^− 1^	2.12 (0.30)	2.05 (0.41)	2.11(0.42)	0.534	0.739	0.263	0.480
Tb. Th, μm	68 (15)	71 (15)	72 (17)	0.244	0.171	0.120	0.946
Tb. Sp, μm	410 (66)	423 (107)	410 (104)	0.677	0.956	0.480	0.424
Inhomogeneity, μm	165 (41)	178 (48)	169 (47)	0.351	0.575	0.149	0.399
Ct. Th, μm	775 (160)	759 (235)	840 (160)	**0.015**	**0.050**	0.245	**0.007**
Ct. Po, %	2.0 (1.46)	2.8 (1.72)	2.2 (1.57)	0.064	0.373	**0.020**	0.157

*Abbreviations: BMD* Bone mineral density, *SpA* Spondyloarthritis, *Ct.* Cortical, *Tb.* Trabecular, *Tb. meta* Bone mineral density peripheral trabecular density adjacent to cortex, *Tb. inn* Bone mineral density central medullary trabecular density, *BV/TV* Trabecular bone volume, *Th* Thickness, *Sp* Separation, *Po* Porosity

Bone geometry, microstructure, and volumetric BMD by high-resolution peripheral quantitative computed tomography at the ultradistal radius. Results are median (IQR)

Bold indicates significant differences (*p* < 0.05)

### Bone microarchitecture and vBMD with respect to glucocorticoid treatment in patients with nr-axSpA

Patients were divided into two groups according to their prior exposure to GC treatment (> 5 mg of prednisolone equivalent over ≥ 3 months) in their medical history. Patients treated with GC showed a reduced trabecular vBMD compared with GC-naïve patients (*p* = 0.044), whereas there was no difference in total or cortical vBMD. Further, especially vBMD of the centrally located trabecular network (*p* = 0.014) showed a reduction, whereas there was no difference of trabecular bone adjacent to cortex (*p* = 0.209). Bone microstructure showed deterioration of the trabecular network with reduced trabecular bone volume (BV/TV; *p* = 0.045) as well as increased inhomogeneity index (*p* = 0.007) and Tb.Sp (*p* = 0.037) in patients treated with prednisolone. Cortical thickness showed no significant difference (*p* = 0.959). (Fig. 2b).

### Factors associated with cortical bone changes in nr-axSpA

The first multiple logistic regression model for demographic variables included sex, age, BMI, and smoking status. Male sex was associated with lower cortical vBMD (*p* = 0.001), whereas female sex was associated with lower trabecular vBMD (*p* <  0.001). The model for total vBMD revealed no demographic predictor (Table 4). In a second model, sex, age, BMI, remission status, disease duration, treatment with TNFi, prior GC treatment, HLA-B27 status, and peripheral arthritis were included. In these models, male sex again was associated with lower cortical vBMD (*p* = 0.004), whereas female sex was associated with lower trabecular vBMD (*p* = 0.001). The model for total vBMD identified no predictors. Prior GC treatment was associated with lower trabecular vBMD (*p* = 0.041). Interestingly, disease duration was positively associated with trabecular vBMD (*p* = 0.010). All further parameters, including age, BMI, remission status, treatment with TNFi, HLA-B27 status, and peripheral arthritis, did not show significant results.

**Table 4:** Predictors of reduced total, cortical, and trabecular bone mineral density in patients with nonradiographic axial spondyloarthritis

	Total BMD			Ct. BMD			Trab. BMD		
	β	T	p	β	T	p	β	T	p
Model 1									
Age	0.006	0.068	0.946	−0.038	−0.449	0.654	−0.073	− 0.952	0.342
Sex (male vs. female)	− 0.143	−1.730	0.086	0.285	3.523	**0.001**	−0.466	−6.324	**< 0.001**
BMI	0.079	0.915	0.362	−0.004	− 0.042	0.966	0.083	1.080	0.282
Smoking (yes/no)	0.118	1.415	0.159	−0.041	− 0.505	0.615	0.103	1.378	0.171
Intercept	–	11.570	**< 0.001**	–	29.784	**< 0.001**	–	12.765	**< 0.001**
*R*^2^ adjusted	–	0.016	**–**	–	0.059	**–**	–	0.219	**–**
Model 2									
Age	−0.109	−0.913	0.364	−0.094	− 0.858	0.393	− 0.151	−1.498	0.138
Sex	−0.095	−0.847	0.399	0.300	2.937	**0.004**	−0.420	−4.448	**< 0.001**
BMI	0.115	1.036	0.303	0.017	0.164	0.870	0.159	1.689	0.095
Remission	−0.017	−0.150	0.881	0.015	0.147	0.883	0.048	0.512	0.610
Disease duration	0.157	1.241	0.218	−0.169	−1.460	0.148	0.280	2.625	**0.010**
Anti-TNF	−0.094	−0.776	0.440	−0.131	−1.176	0.243	−0.160	−1.557	0.123
GC (yes/no)	−0.132	−1.131	0.261	0.109	1.023	0.309	−0.205	−2.078	**0.041**
HLA-B27 positivity	−0.027	−0.254	0.800	0.002	0.020	0.984	0.027	0.304	0.762
Peripheral arthritis	0.011	0.095	0.924	−0.041	−0.404	0.687	−0.093	−0.987	0.327
Intercept	–	8.522	**< 0.001**	–	23.225	**< 0.001**	–	9.761	**< 0.001**
*R*^2^ adjusted	–	−0.038	–	–	0.130	–	–	0.258	–

*Abbreviations: BMD* Bone mineral density, *Ct. BMD* Cortical bone mineral density, *Trab. BMD* Trabecular bone mineral density, *axSpA* axial spondyloarthritis, *BMI* Body mass index, *Anti-TNF* Current treatment with anti-tumor necrosis factor α inhibitor, *GC* History of glucocorticoid treatment with ≥ 5 mg prednisolone equivalent daily for > 3 mo

Remission status according to Ankylosing Spondylitis Disease Activity Score (ASDAS-CRP, inactive disease ASDAS-CRP < 1.3)

Bold indicates significant differences (*p* < 0.05)

## Discussion

Bone loss is a well-known phenomenon in axSpA, especially in long-standing disease. In the present study, we performed a detailed analysis of bone microstructure in a large cohort of patients with axSpA, with disease duration of less than 2 years in nearly 50% of patients. Our analysis shows that patients with nr-axSpA are characterized by a virtually exclusive cortical but not trabecular bone pathology, which is remarkable. Cortical bone changes characterized by changed geometry, BMD, and microstructure were found early in the disease course of axSpA.

To date, different structural and compartmental changes in bone have been identified using HR-pQCT in systemic inflammatory diseases. Whereas in rheumatoid arthritis a significant deterioration of cortical and trabecular bone has been described, especially in ACPA-positive patients, patients with psoriatic arthritis show predominantly changes of trabecular bone [27, 28]. In contrast, in patients with IBD, primarily a loss of cortical bone has been found [29]. The described changes of cortical bone in nr-axSpA reflect bone structural changes found in patients with IBD.

Our study shows that cortical bone loss as evidenced by cortical thinning happens early in axSpA and can already be found within the first 2 years of disease. In accordance with this, a previous HR-pQCT study of male patients with established AS showed a reduction of cortical vBMD and increased cortical porosity [24]. In this study, however, patients had long-standing disease, with a median duration of symptoms longer than 20 years. Cortical thinning and low cross-sectional area in the peripheral skeleton in patients with AS were strongly associated with the presence of vertebral fractures [24]. These findings are in accordance with the previously described association of cortical thinning and loss of cortical BMD at the radius and the tibia with the occurrence of vertebral fractures in men in the general population [30]. The median disease duration in our study was much shorter (6.5 years) than in the aforementioned study. Nonetheless, about half of the patients with nr-axSpA did not have early disease (< 2 years) anymore and were treated with TNFi. The fact that some patients had a disease duration longer than 2 years may also explain the observation that 7% of the patients with axSPA already had a history of low traumatic fracture. Overall, however, our data show systemic deterioration of cortical bone microstructure even at an early stage of disease in patients with nr-axSpA.

Data are so far limited regarding the factors that influence bone microarchitecture in patients with nr-axSpA and patients with AS. Multiple logistic regression identified male sex to be associated with lower cortical vBMD. Interestingly, loss of cortical vBMD was independent of standard disease-related features such as age, BMI, remission status, anti-TNF treatment, HLA-B27 status, and peripheral arthritis. In contrast to cortical vBMD, trabecular vBMD was lower in women than in men. These findings are in accordance with previous population-based studies showing higher trabecular vBMD in men, whereas cortical vBMD is higher in women [31]. Disease duration was associated positively with higher trabecular vBMD in the present study, which can be explained by longer disease duration in men than in women. These results confirm previous findings of Haroon et al., who investigated sex differences among patients with AS and showed decreased cortical vBMD in men, whereas trabecular vBMD was worse in women [23].

Although nr-axSpA specifically affects cortical bone, an evaluation of those patients treated with GCs > 5 mg daily for > 3 months in the past showed specific loss of trabecular bone associated with reduction of trabecular vBMD, lower total bone volume, and higher trabecular separation and inhomogeneity. Sutter et al. investigated postmenopausal women treated with oral GCs for > 3 months and age-/race-matched control subjects using HR-pQCT and DXA. In their study, despite no difference in areal BMD, GC-treated women showed an impairment of cortical and trabecular vBMD and bone microarchitecture [32]. Moreover, later stages of the disease, particularly in patients with severe AS with syndesmophyte formation, also trabecular vBMD appears to decrease, which is reflected by a study from Devogelaer and colleagues [9].

The strength of the present study is a detailed assessment of bone macro- and microarchitecture using HR-pQCT in patients with axSpA. To date, this is the largest nr-axSpA cohort with detailed bone analysis by HR-pQCT. A further strength and novelty of this work is the assessment of patients with short disease duration. A limitation is, of course, that HR-pQCT cannot analyze the spine but is confined to peripheral sites. However, several studies have previously shown that structural changes in the peripheral bones reflect bone changes as well as fracture risk in the axial skeleton [22, 23].

The reason why nr-axSpA virtually exclusively affects cortical bone is not entirely clear. It can be speculated that such changes reflect increased cortical bone remodeling. For instance, it has long been known that the remodeling of cortical bone is highly dependent on strain [33], and later concepts even suggested that cortical bone remodeling may even be entirely dependent on microcracks [34]. Hence, altered bone responses to mechanical forces in nr-axSpA may be triggers for increased cortical bone remodeling and bone loss. In accordance with this, prostaglandin E_2_, a prototype inflammatory mediator released upon injury and having a central role in nr-axSpA, has been shown to enhance cortical bone remodeling [35]. The disbalance in cortical bone in patients with nr-axSpA, however, may stem from cytokines such as interleukin-17, which effectively suppress bone formation but at the same time increase bone resorption [36, 37].

## Conclusions

In this study, we show that axSpA specifically leads to an early and virtually exclusive loss of cortical bone, thereby contrasting with many other inflammatory diseases but resembling features of bone loss observed in patients with IBD. In contrast, trabecular bone does not appear to be directly influenced by axSpA.

## Additional file

Additional file 1: **Table S1** Demographic parameters, disease-specific characteristics, and bone microstructure in patients with nonradiographic axial spondyloarthritis (nr-axSpA) with or without TNFi treatment. (DOCX 16 kb)

## Abbreviations

ACPA: Anticitrullinated protein antibodies
AS: Ankylosing spondylitis
ASAS: Assessment of Spondyloarthritis international Society
ASDAS: Ankylosing Spondylitis Disease Activity Score
BASDAI: Bath Ankylosing Spondylitis Disease Activity Index
BASMI: Bath Ankylosing Spondylitis Metrology Index
BMD: Bone mineral density
BMI: Body mass index
BV/TV: Bone volume per tissue volume
CRP: C-reactive protein
Ct: Cortical bone
Ct.Po: Cortical porosity
Ct.Th: Cortical thickness
DMARD: Disease-modifying antirheumatic drug
DXA: Dual-energy x-ray absorptiometry
ESR: Erythrocyte sedimentation rate
GC: Glucocorticoid
HA: Hydroxyapatite
HLA-B27: Human leukocyte antigen B27
HR-pQCT: High-resolution quantitative computed tomography
IBD: Inflammatory bowel disease
MASES: Maastricht Ankylosing Spondylitis Enthesitis Score
SpA: Spondyloarthritis
Tb: Trabecular bone
Tb.Sp: Trabecular separation
TNF: Tumor necrosis factor α
TNFi: Inhibition of tumor necrosis factor α
vBMD: Volumetric bone mineral density
