# Supplementary material for: Cortical bone loss is an early feature of nonradiographic axial spondyloarthritis
Source: Arthritis Res Ther. 2018 Aug 30;20:202. doi: 10.1186/s13075-018-1620-1 (PMC6117894; doi:10.1186/s13075-018-1620-1)
Supplement: Supplementary file 1 — Table S1 Demographic parameters, disease-specific characteristics, and bone microstructure in patients with nonradiographic axial spondyloarthritis (nr-axSpA) with or without TNFi treatment. (DOCX 16 kb) [file 13075_2018_1620_MOESM1_ESM.docx]

Additional file 1: **Table S1**. Demographic parameters, disease specific characteristics and bone micro-structure in non-radiographic axial spondyloarthritis (nr-axSpA) patients with or without TNF inhibitor treatment

|  | Anti-TNF treatment | | p-value |
| --- | --- | --- | --- |
|  | yes (N=59) | no (N=42) |  |
| **Demographic characteristics** |  |  |  |
| Age (years) | 45.0 (15) | 46.0 (17) | 0.610 |
| Females, n (%) | 17 (28.8) | 25 (59.5) | **0.002** |
| BMI (kg/m2) | 25.0 (9) | 25.0 (5.3) | 0.462 |
| Height (m) | 1.74 (0.1) | 1.74 (0.1) | 0.381 |
| Weight (kg) | 83.0 (28) | 79.0 (16) | 0.128 |
| Disease Duration (years) | 5 (9) | 1 (4) | **0.001** |
| **Bone Geometry** |  |  |  |
| Total Bone Area (mm^2^) | 341 (112) | 305 (104) | **0.048** |
| Ct. Area (mm^2^) | 60 (23) | 58 (18) | 0.657 |
| Tb. Area (mm^2^) | 267 (96) | 245 (82) | 0.087 |
| **Volumetric Bone Mineral Density** |  |  |  |
| Total BMD (HA/cm^3^) | 312 (75) | 314 (60) | 0.772 |
| Ct. BMD (HA/cm^3^) | 809 (77) | 836 (56) | **0.043** |
| Tb. BMD (HA/cm^3^) | 181 (47) | 173 (43) | 0.457 |
| Tb. meta BMD (HA/cm^3^) | 240 (42) | 231 (47) | 0.615 |
| Tb. inn BMD (HA/cm^3^) | 141 (51) | 132 (46) | 0.451 |
| **Bone Microstructure** |  |  |  |
| BV/TV (%) | 15.1 (3.9) | 14.4 (3.6) | 0.455 |
| Tb. N (mm^-1^) | 2.11 (0.31) | 2.01 (0.37) | 0.385 |
| Tb. Th (μm) | 71 (16) | 70 (14) | 0.831 |
| Tb. Sp (μm) | 399 (64) | 422 (93) | 0.436 |
| Inhomogeneity (μm) | 165 (44) | 170 (47) | 0.970 |
| Ct. Th (μm) | 765 (248) | 765 (153) | 0.639 |
| Ct. Po (%) | 2.25 (1.79) | 2.06 (1.29) | 0.077 |
